# Folic Acid Antagonists: Antimicrobial and Immunomodulating Mechanisms and Applications

**DOI:** 10.3390/ijms20204996

**Published:** 2019-10-09

**Authors:** Daniel Fernández-Villa, Maria Rosa Aguilar, Luis Rojo

**Affiliations:** 1 Instituto de Ciencia y Tecnología de Polímeros, Consejo Superior de Investigaciones Científicas, CSIC, 28006 Madrid, Spain; dafer-vi@hotmail.com (D.F.-V.); mraguilar@ictp.csic.es (M.R.A.); 2 Consorcio Centro de Investigación Biomédica en Red de Bioingeniería, Biomateriales y Nanomedicina, 28029 Madrid, Spain

**Keywords:** folic acid antagonists, antifolates, antibiotics, antibacterials, immunomodulation, sulfonamides, antimalarial

## Abstract

Bacterial, protozoan and other microbial infections share an accelerated metabolic rate. In order to ensure a proper functioning of cell replication and proteins and nucleic acids synthesis processes, folate metabolism rate is also increased in these cases. For this reason, folic acid antagonists have been used since their discovery to treat different kinds of microbial infections, taking advantage of this metabolic difference when compared with human cells. However, resistances to these compounds have emerged since then and only combined therapies are currently used in clinic. In addition, some of these compounds have been found to have an immunomodulatory behavior that allows clinicians using them as anti-inflammatory or immunosuppressive drugs. Therefore, the aim of this review is to provide an updated state-of-the-art on the use of antifolates as antibacterial and immunomodulating agents in the clinical setting, as well as to present their action mechanisms and currently investigated biomedical applications.

## 1. Introduction to Folic Acid Antagonists: Antifolates

**Figure 1:**
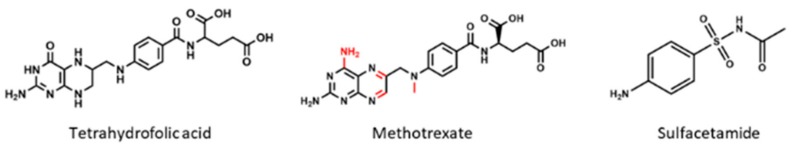
Chemical structures of tetrahydrofolic acid and two antifolates: methotrexate (classical antifolate) and sulfacetamide (non-classical antifolate), commonly used as folic acid antagonists. Differences between tetrahydrofolate molecule (THF) and methotrexate are pointed out in red.

In order to comprehend the use of antifolates as therapeutic agents, it is necessary to focus on the central molecule of their metabolism: folic acid. This metabolite belongs to the B-vitamins family, a group of small water soluble molecules, which act as enzymatic cofactors to carry out diverse metabolic functions [1]. More precisely, the term “folic acid” or “folate” is usually employed when referring to the tetrahydrofolate molecule (THF), which is composed of a pteridine portion, a *p*-aminobenzoic acid (PABA) and a L-glutamate residue (Figure 1). However, due to its role as a one-carbon donor, its structure may present some subtle modifications depending on the carbon transfer reactions where they participate, thus comprising more than 150 molecules under this term [1,2].

Furthermore, folate derivatives are subject of polyglutamation. In humans, the predominant form of folate in plasma is monoglutamate, while within cells is the pentapolyglutamate derivative (five glutamate residues) [3]. The addition of glutamate residues at the γ -carboxyl moiety is mediated by the folylpoly-γ-glutamate synthetase (FPGS) and confers to the molecules an increase in negative charge that results in their intracellular accumulation for longer periods of time. It is important to note that polyglutamated forms have higher affinity for most of the folate-dependent enzymes when compared with monoglutamate folate derivatives and this also happens with their structurally similar inhibitors [1,4].

Regarding its function, folic acid metabolism is essential to ensure the proper functioning of all living cells [5,6]. Folate takes part in numerous reactions, which relate to many biosynthetic pathways that allow the correct synthesis of proteins and nucleic acids in the different organisms. On the one hand, it participates in the reactions of synthesis of some amino acids such as methionine, serine, glycine, histidine or glutamate and in the formylation of transference RNAs, essential steps for protein synthesis. On the other hand, it is also involved in both purine and pyrimidine nitrogenous bases syntheses and it epigenetically regulates gene expression via previously mentioned one-carbon transfer reactions [1,2]. For these reasons, folic acid supplementation is of special importance during pregnancy and regenerative processes [6,7].

All these metabolic functions that are essential for cell survival highlight the importance of molecules with an antagonistic effect: the antifolates. These metabolites have been historically classified in two groups: the ones which have a structural similarity with folic acid, or “classical antifolates”, and which compete with various enzymes, slowing down or even inhibiting folic acid’s metabolism; and the ones which affect folic acid biosynthesis, also known as “non-classical antifolates”, and which structurally diverge from folate (Figure 1) [4].

As it will be commented throughout this review, these structural differences have been utilized since the very beginning for their use as antibiotics, exploiting the metabolic differences between humans and microorganisms. For instance, humans are not able to synthesize folate de novo, thus having to ingest it dietary. On the contrary, bacteria do possess specific enzymes that allow its de novo synthesis while their cell walls are impermeable to it and its derivatives because they lack the necessary folate receptors that internalize the different derivatives. For this reason, inhibiting specific enzymes that participate at folate biosynthesis was one of the first methods to cope with bacterial infections already in the 1930s [8].

In this review, we have summarized the fundamentals of the use of antifolates in clinic as antibacterial, antimalarial and immunomodulating agents, focusing on their action mechanisms, spectra of activity, indications, adverse effects and the emerging resistances which have appeared over time and which have led to look for new alternatives. Furthermore, special emphasis has been made on possible biomedical applications, which are being recently investigated when applicable.

## 2. Mechanisms of Action of Antifolates

**Figure 2:**
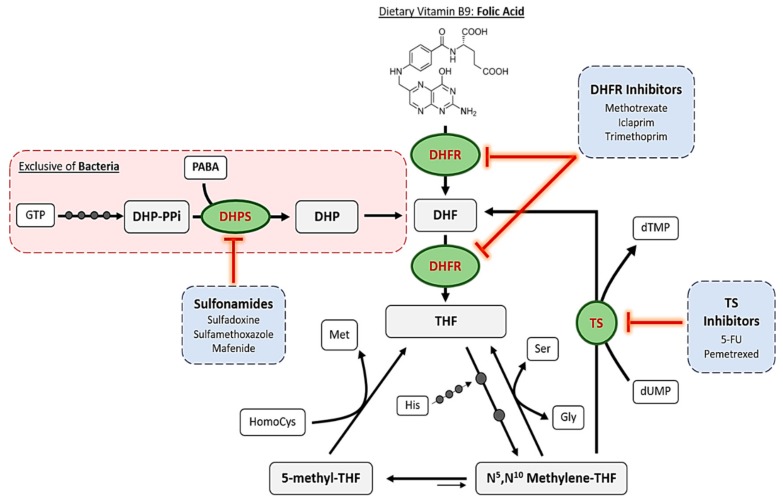
Summarized pathway of folic acid metabolism, including bacterial de novo synthesis, reduction and TS-mediated feedback loop. Principal enzymes targeted by antifolates are highlighted in green circles. Examples of the different inhibitors are listed in the blue boxes. Small dark grey circles over the arrows indicate an enzymatic reaction. Abbreviations (Abbs.): DHP = dihydropteroate, DHP-PPi = dihydropteroate pyrophosphate, DHPS = dihydropteroate synthase, DHF = dihydrofolate, DHFR = dihydrofolate reductase, Gly = glycine, GTP = guanosine triphosphate, His = histidine, HomoCys = homocysteine, Met = methionine, PABA = p-aminobenzoic acid, Ser = serine, THF = tetrahydrofolate, and TS = thymidylate synthase.

The mechanism of action of antifolates depends on the enzyme where they act. Although numerous enzymes contribute to folate metabolism, some of them highlight for their relevant role in folate’s historical use as therapeutic compounds. Figure 2 schematizes these principal enzymes, the pathways where they are involved and some of the compounds, which inhibit them.

### 2.1. Dihydropteroate Synthase

**Figure 3:**
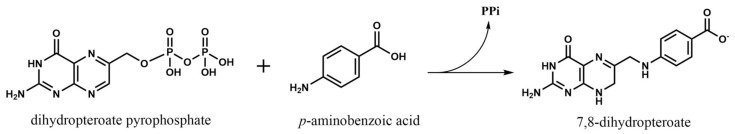
Biosynthesis reaction of 7,8-dihydropteroate catalyzed by dihydropteroate synthase.

The first, and one of the most targeted enzymes in folate metabolism has been dihydropteroate synthase (DHPS, EC 2.5.1.15). This is considered as the first regulation point at the de novo folate biosynthesis, which is exclusive of bacteria. This enzyme is encoded by *folP* gene and catalyzes the synthesis reaction of dihydropteroate, the immediate precursor to dihydrofolate, which is next reduced to THF, adding a PABA molecule to a dihydropteroate pyrophosphate (DHP-PPi) and releasing the pyrophosphate moiety (PPi) (Figure 3) [4].

Sulfonamides belong to the non-classical antifolates group and are the ones that inhibit DHPS by penetrating into the PABA pocket of the enzyme, avoiding the entrance of PABA to the reaction site and forming an analog that cannot be used as a subtract in the following reaction of the folate cycle [8]. Thus, they are competitive inhibitors of this enzyme and they cause a drastic reduction of folate levels. As bacteria cannot internalize exogenous folate, this reduction leads to errors in DNA synthesis because of thymine depletion, a cell death mechanism which was defined as “thymineless death” [9].

Chemically, they are defined as the amides of sulfonic acids and are classified attending IUPAC’s nomenclature for amides in primary, secondary or tertiary, depending on their number of substituents, which could be diverse. In fact, sulfonamides are ranked in the 22nd position of the list of most frequent side chains present in known drugs elaborated by Bemis and Murcko [10,11,12]. On the one hand, this great tunability among compounds has allowed us to have available many similar drugs with different potencies, cytotoxicities or pharmacokinetic properties and, moreover, it has contributed to spread their use not only as antibiotics but also as treatments for complex diseases such as Alzheimer, psychosis and many types of cancer [8,13,14]. Nevertheless, it has also led to the appearance of bacterial drug resistances, as it will be addressed later.

### 2.2. Dihydrofolate Reductase

**Figure 4:**
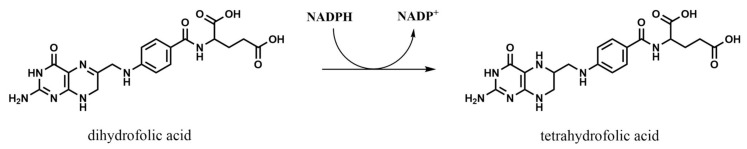
Reduction reaction of dihydrofolate to tetrahydrofolate catalyzed by dihydrofolate reductase.

Dihydrofolate reductase (DHFR, EC 1.5.1.3) is the most studied enzyme in folate pathway due to its relevance in the maintenance of the cycle. Reduction of dihydrofolate (DHF) ensures an intracellular pool of different THF derivatives that are used in various one-carbon transference reactions and biosynthetic processes. The general reaction, which consumes NADPH, is schematized in Figure 4, although it accepts modifications depending on the substituents of the DHF utilized as substrate [15]. After this reaction, polyglutamation by FGPS takes place in order to accumulate the final products inside the cells.

As demonstrated by Stone and Morrison, classical inhibitors of DHFR follow a model of competitive inhibition with respect to DHF, except folinate which acts as a competitive antagonist of NADPH and as a noncompetitive antagonist of DHF [16]. However, they all lead to cell death by THF pool depletion.

Along the years, DHFR structures from many organisms have been elucidated by crystallography, not only for its interest as a target for antibacterial and antiprotozoal drugs but also because human DHFR is a target for immunosuppressors and cytostatic agents. In fact, only trimethoprim, which was the first antibacterial DHFR inhibitor, is used nowadays as part of a combination therapy with a DHPS inhibitor (sulfamethoxazole) with antibacterial purposes [8]. Thus, the majority of commercialized DHFR inhibitors are administered for treating different types of cancer, autoimmune diseases and protozoal infections such as malaria or toxoplasmosis [17].

### 2.3. Thymidylate Synthase

**Figure 5:**
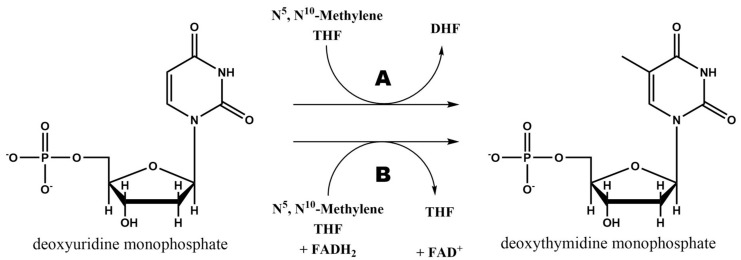
dTMP biosynthesis reaction from deoxyuridine-5′-monophosphate (dUMP) and a THF derivative catalyzed by thymidylate synthase (**A**) and flavin-dependent thymidylate synthase (**B**).

Folate pathway is linked to pyrimidine synthesis via thymidylate synthase (TS) in order to provide new DHF to the cycle. This enzyme uses N^5^,N^10^-Methylene THF to methylate 2′-deoxyuridine-5′-monophosphate (dUMP) and synthesize 2′-deoxythymidine-5′-monophosphate (dTMP) [8]. The general reaction catalyzed by this enzyme is schematized in Figure 5A.

As it can be observed in Figure 5B, there is a second mechanism to synthesize dTMP from dUMP and N^5^,N^10^-Methylene THF. Although the vast majority of organisms use the TS encoded by *thy*A gene (or *TYMS* in humans), some bacteria and archaea use a flavin-dependent TS (FDTS) encoded by *thy*X gene, which is the responsible of this second reaction. These organisms lack of DHFR and thymidine kinase too, but can survive in environments where there is no free thymidine, showing a different approach for thymine synthesis [8,18].

There are several differences between the two enzymes, their catalysis mechanisms and reaction steps, and these differences are what makes FDTS-organisms resistant to conventional antifolates. Among these organisms, many pathogenic ones are found such as *Mycobacterium tuberculosis*, *Helicobacter pylori* or *Clostridium difficile*. For this reason new drugs are being synthesized since the past few years to target FDTS, taking advantage of the differential binding modes of the substrates when compared with normal TS enzyme [19,20,21].

Furthermore, FDTS is not present in humans and this makes it a suitable target for reducing the associated harmful side effects. In contrast, *thyA*-encoded TSs do exhibit a great structural similarity when comparing bacterial and human enzymes and this has led to the failure of its inhibitors as antibacterial drugs due to the associated toxicity. Nevertheless, TS is still today a great target in many types of cancer where uracil metabolism is accelerated, increasing their selectivity and tolerance. Classical inhibitors of this enzyme such as raltitrexed act as competitive antagonist of N^5^,N^10^-Methylene THF, finally leading to thymine depletion. On the other hand, non-classical inhibitors like 5-fluorouracil irreversibly inhibit TS but also interact with RNAs, altering the normal functions of the cells too [8].

### 2.4. Other Enzymes

Although DHPS, DHFR and TS are the principal and most studied enzymes involved in folate pathway, there are other enzymes which stand out for their relevance at certain biomedical applications.

#### 2.4.1. Bifunctional DHFR-TS

In humans, the DHFR and TS activities are well separated, being carried out by totally different enzymes. However, some protozoal parasites such as *Plasmodium falciparum* (malaria), *Leishmania major* (leishmaniasis), *Toxoplasma gondii* (toxoplasmosis) or *Trypanosoma cruzi* (Chagas’ disease) possess a bifunctional DHFR-TS encoded by a single gene. In these cases, the product from the first reaction (DHF from TS reaction) is directed towards the active site of the DHFR domain where reduction to THF takes place [22]. This unique conformation and the process of substrate channeling between subunits makes suitable the development of novel specific inhibitors for this enzyme [23].

#### 2.4.2. Purine Synthetic Pathway

Purines biosynthesis is also a THF-dependent process necessary for DNA synthesis. N^10^-Formyl-THF acts as a cofactor in two reactions of the pathway, which are catalyzed by glycinamide ribonucleotide formyltransferase (GARFT, EC 2.1.2.2) and the aminoimidazole-4-carboxamide ribonucleotide formyltransferase (AICARFT, EC 2.1.2.3) domain of the bifunctional aminoimidazole-4-carboxamide ribonucleotide formyltransferase/inosine monophosphate cyclohydrolase (ATIC) enzyme [24]. Thus, the use of certain antifolates as inhibitors of these two enzymes have been demonstrated to be another way to disrupt the normal DNA synthesis in highly multiplying cells. Some non-classical antifolates have been synthesized and successfully inhibit tumor growth both in vitro and in vivo [25,26]. However, this approach cannot be applied for *P. falciparum* infections because it lacks of the necessary enzymes for the de novo purine synthesis [27].

#### 2.4.3. Folylpoly-γ-Glutamate Synthetase

Folylpoly-γ-glutamate synthetase (FPGS, EC 6.3.2.17) is a pivotal enzyme which acts as the central regulator of folate cycle by adding glutamate residues to the different folate and classical antifolate derivatives. It acts in coordination with γ-glutamyl hydrolase (GGH, EC 3.4.19.9) that catalyzes the inverse reaction to produce the monoglutamate forms that exit the cell via ATP-binding cassette transporters [28]. Polymorphisms on their corresponding genes have been found to produce enzymes with lower and higher expressions, respectively, that correlate with resistance to antifolates as chemotherapeutic agents, indicating their suitability as antifolate targets [29]. In addition, regarding *P. falciparum*, a bifunctional dihydrofolate synthase/folylpoly-γ-glutamate synthetase enzyme (DHFS-FPGS) has been reported, opening a new target for antimalarial drugs development [30].

## 3. Antifolates as Antibiotic Agents

As it was commented above, the fact that classic antifolates are considerably negatively charged molecules and, consequently, they are unable to diffuse across bacterial membranes, has limited their use as antibacterials. That is the reason why almost all commercialized antibacterial antifolates belong to the non-classical group, more precisely, to the sulfonamides family, having bacterial DHPS as their target, which is not present in humans, and thus having less side effects.

Table 1 summarizes the most important antifolates used in clinical setting. They are commercialized nowadays even though some resistances might have developed over time, and some of them are used both as antibacterials and antiparasitic agents.

### 3.1. Mafenide Acetate

**Figure 6:**
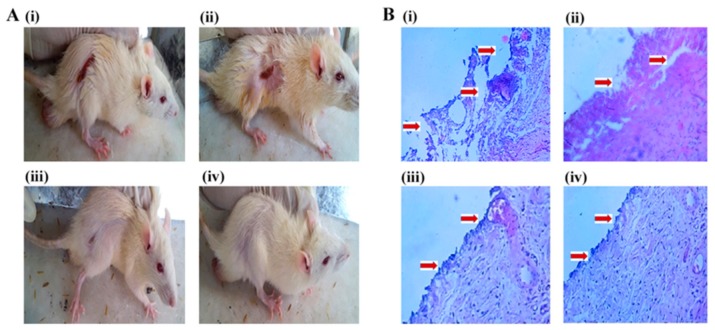
(**A**) Comparative burn wound healing images of rats after 21 days. (**B**) Histopathology of re-epithelialized rat skin after 21 days treatment, at 10× magnification. The treatment group includes (i) untreated (diseased control); (ii) silver sulfadiazine marketed cream; (iii) Silver sulfadiazine-loaded solid lipid nanoparticles-containing chitosan gel; (iv) Silver sulfadiazine-loaded solid lipid nanoparticles and DNase-I-containing chitosan gel. Reproduced from Patel et al. [41].

4-(aminomethyl)benzenesulfonamide, also called mafenide acetate, or just mafenide, is a synthetic drug, chemically related to sulfonamides and PABA, which has a methylene group between the benzene ring and the amino nitrogen (Figure 6). However, this subtle change in its conformation seems to completely change its action mechanism. Mafenide is not antagonized by PABA like the rest of sulfonamides, meaning that they not compete for the same binding site on bacterial DHPS [31]. Although this was confirmed 30 years ago, the bactericidal mode of action of this compound is still unclear [32].

After a severe burn, the wound becomes the perfect environment for bacterial infections as there is no epidermal protection. Mafenide is mainly administered as creams over those wounds to reduce the bacteria present in burnt tissue and to promote its regeneration [33]. Nevertheless, this secondary outcome was under discussion for some time and now it has been reported that mafenide displays some cytotoxicity when applied at the high concentrations at which it is administered. Although its cytotoxic mechanism has not been completely elucidated, it seems that mafenide inhibits the de novo base synthesis by altering hormones concentrations, folate receptors expressions and/or the pH in the wound. Therefore, while protecting from many infectious bacteria, mafenide delays wound healing and reduces the breaking strength of the already healed wounds [34].

Mafenide is active against a wide range of Gram-positive and Gram-negative bacteria, including *Clostridium* spp. and *Pseudomonas aeruginosa*, specially problematic pathogens which invade burn wounds and which have high levels of mortality [33,35]. In fact, it is the most active agent overall against Gram-negatives. In contrast, mafenide is not active against yeasts, which finally would lead to their overgrowth in the burn wound if applied alone. To solve this, it is usually co-administered with nystatin or miconazole [33].

Mafenide is considered as a first line treatment for severe burns and is of particular importance for treating silver sulfadiazine-resistant *Pseudomonas* spp [36]. In comparison with this other antifolate (discussed in the next epigraph), mafenide has a greater tissue penetration as it is absorbed through devascularized tissue and eschars, being the drug choice for third- and fourth-degree burns and burns that reach joints, bones or tendons [37].

However, there are some clinical problems associated to its use. After the application, mafenide is metabolized to a carbonic anhydrase inhibitor which competes with *p*-nitrophenyl acetate for binding to the active site of the enzyme and this could lead to systemic metabolic acidosis when high quantities are applied [33]. Furthermore, the European Chemicals Agency suspects that mafenide could be persistent and hazardous to the aquatic environment and a skin irritant, mutagenic toxicant [38].

### 3.2. Silver Sulfadiazine

Silver sulfadiazine is by far the most administered topical antifolate and antimicrobial agent for severe burn treatments, due to its good coverage against pathogens, easy and painless administration and few related side effects. It is a combination of sodium sulfadiazine and silver nitrate, where silver gets complexed to propylene glycol, isopropyl myrisolate and stearyl alcohol. It is commercialized as creams containing 1% of the insoluble compound in micronized form for topical administration and, compared to mafenide acetate, its application is not painful produces a cooling, soothing sensation [33].

The compound exhibits a broad antimicrobial activity, being bactericidal for many Gram-negative (like *Pseudomonas* spp.) and Gram-positive bacteria. In addition, another advantage compared to mafenide is its effectivity against yeasts (such as *Candida albicans*), not needing additional fungicides. This antibiotic effect is due to the synergistic action of each component. On the one hand, silver binds to some amino acids, denaturating proteins, as well as to surface membranes, causing proton leaks that ultimately lead to cell death by a non-specific biocide mechanism. On the other hand, sulfadiazine moiety acts like a typical sulfonamide, inhibiting bacterial DHPS and affecting folic acid synthesis [39].

Regarding its cytotoxicity, silver sulfadiazine is a relatively safe compound. In contrast to mafenide, it does not inhibit carbonic anhydrase, thus being useful in situations where such agent is contraindicated. Mild cutaneous eruptions may appear causing allergic reactions in patients with sulfa allergies. A brief silver sulfadiazine-caused leukopenia which appears a few days after the burn has been deeply studied too. However, the current general opinion attributes it to a leukocyte margination to the wound instead of a bone marrow suppression since it resolves spontaneously [32,33].

The major clinical problem associated to silver sulfadiazine is the impairment on re-epithelization that it produces and its cytotoxicity against fibroblasts. Therefore, it may not be recommended for partial-thickness burns. Nevertheless, due to their numerous advantages, research is being carried out to solve these inconveniences. Nalbandi & Amiri designed polyvinyl alcohol-based nanofibers loaded with silver sulfadiazine/cyclodextrin nanocapsules in order to achieve a controlled release. They demonstrated that the resulting nanofibers had an increased antimicrobial activity due to an enhanced particle surface reactivity which damage the bacterial cell walls [40].

In other study, Patel et al. developed a chitosan gel loaded with solid lipid nanoparticles of silver sulfadiazine and supplemented with deoxyribonuclease-I. The combination resulted in lesser toxicity against dermal fibroblasts thanks to the drug-controlled release from the nanoparticles, while it maintained the minimal inhibitory concentration for an extended time. In addition, deoxyribonuclease-I was demonstrated to disrupt the extracellular DNA of the biofilm increasing the action range of silver sulfadiazine. In vivo tests showed an acceleration on wound healing and complete re-epithelization of skin, thus confirming the superiority of the proposed therapy when compared with the current treatments [41].

New strategies for wound regenerations were reviewed by Vigani et al. Her group developed silver sulfadiazine-loaded chitosan/montmorillonite nanocomposites for the topical treatment of chronic skin wounds. Those systems combined the antimicrobial activity of the sulfonamide with the regenerative properties of chitosan and montmorillonite, reducing fibroblasts and keratinocytes cytotoxicities and promoting the wound healing process. Moreover, analogously to Nalbandi et al., drug loading into nanocomposites enhanced the antibiotic properties, especially against *P. aeruginosa* [42].

### 3.3. Sulfadiazine

Sulfadiazine (2-sulfanilamidopyrimidine), without the silver moiety, is used mainly for treating urinary tract infections and as a treatment for some parasitic diseases, including malaria and toxoplasmosis. Regarding its bactericidal function, sulfadiazine exhibits a broad-spectrum activity against most Gram-positive and Gram-negative bacteria by targeting the DHPS, as the rest of sulfonamides [43].

Nowadays, with the development of resistances and the availability of other antibiotics, sulfadiazine has lost relevance. Something similar happened when silver sulfadiazine was first marketed for the treatment of burn wounds. In the 1940s, a combination of sulfadiazine and sulfathiazole was recommended for this indication. However, the addition of the silver moiety led to a change in the golden standard, although the tissue penetration considerably decreased. Interestingly, although there is no much recent research on this drug, a few months ago Kurowska et al. developed a novel foam for treating second degree burn wounds containing pectin capped green nanosilver and sulfadiazine, which represents an alternative formulation with an increased penetration, efficacy and easy application, using sulfadiazine instead of silver sulfadiazine [44].

Sulfadiazine has been prescribed also for treating bacterial infections such as encephalitis, otitis media and severe meningococcal meningitis as well as a prophylactic treatment for rheumatic fevers. In contrast to the above-mentioned article where the application was directly on the wound burns, sulfadiazine is orally administered in tablets for the rest of the indications and it is usually well-tolerated [43].

Apart from the possible hypersensitivities to sulfonamides exposure, gastrointestinal upset is one of the most common side effects. However, more serious complications may appear such as encephalopathies or renal failure. In HIV-positive patients it has been described the appearance of sulfadiazine-associated urinary calculi [45]. In addition, most commonly used sulfonamides have been demonstrated to cross the placenta and enter foetal circulation [46]. Regarding sulfadiazine, it is considered a pregnancy category C drug, meaning that animal reproductions studies have shown an adverse effect on the foetus but there are no adequate studies in humans. However, it may be recommended to prevent toxoplasmosis of the foetus or for the maternal treatment of *T. gondi*-caused encephalitis, trying to avoid its use during the first trimester [47].

Regarding current research panorama on this drug, it seems that sulfadiazine will be used as a model scaffold to develop novel sulfadiazine derivatives with new pharmacokinetic properties. In this context, novel sulfadiazine salicylaldehyde-based Schiff bases have been synthesized and characterized. Krátký et al. demonstrated that some of these derivatives were more advantageous than its precursor, being the dehalogenation of the salicylic acid moiety a method that increased the antibacterial and antifungal activities [48]. Moreover, sulfadiazine has also been used to develop novel derivatives which do not inhibit bacterial DHPS but the jack bean urease. This enzyme is the responsible of the stomach disease and pectic ulcers caused by *H. pylori*, among other disturbances. Channar et al. demonstrated that their newly-synthesized drugs inhibited the enzyme by different mechanisms and presented an excellent radical scavenging potency when compared with vitamin C [49].

### 3.4. Sulfacetamide

Sulfacetamide (N-((*p*-aminophenyl)sulfonyl)acetamide) is mainly used for treating ocular infections such as conjunctivitis or trachoma and skin infections like acne vulgaris, rosacea or seborrheic dermatitis. Therefore, most of the products including this drug (usually in form of sodium sulfacetamide) are marketed as creams for topical use or as solutions for ophthalmic administration. It is also commercialized as intravaginal preparations for chlamydial and bacterial vaginitis [50,51]. In all these situations, many pathogens may be involved, thus broad-spectrum antimicrobial compounds like sulfacetamide are highly desirable.

Sulfacetamide is effective against several Gram-positive and Gram-negative bacteria. Regarding its dermatological applications, its effectiveness against *Propionibacterium acnes* must be highlighted. 10% sulfacetamide is usually co-administered with 5% sulfur because of the known antibacterial and anti-inflammatory properties of the first one and the nonspecific antifungal, antibacterial, antiparasitic and keratolytic effects of the last one [50]. While its antibacterial mechanism of action is well-stablished (inhibiting bacterial DHPS like most sulfonamides), there is no data analyzing the molecular mechanism underlying its anti-inflammatory properties.

Attending to its ocular administration, sulfacetamide may be less effective when compared with other drugs for treating conjunctivitis such as neomycin. However, it produces less side effects too [52]. Regarding its associated clinical problems, when topically applied, some mild reactions may appear such as dryness, erythema or pruritus while when ophthalmically used it may irritate the conjunctiva or cause some stinging in younger patients [53]. Furthermore, it has been tried to diminished sulfur’s characteristic odor by adding masking fragrances and designing novel “wash-on–wash-off” formulations to make it more user-friendly [54].

**Figure 7:**
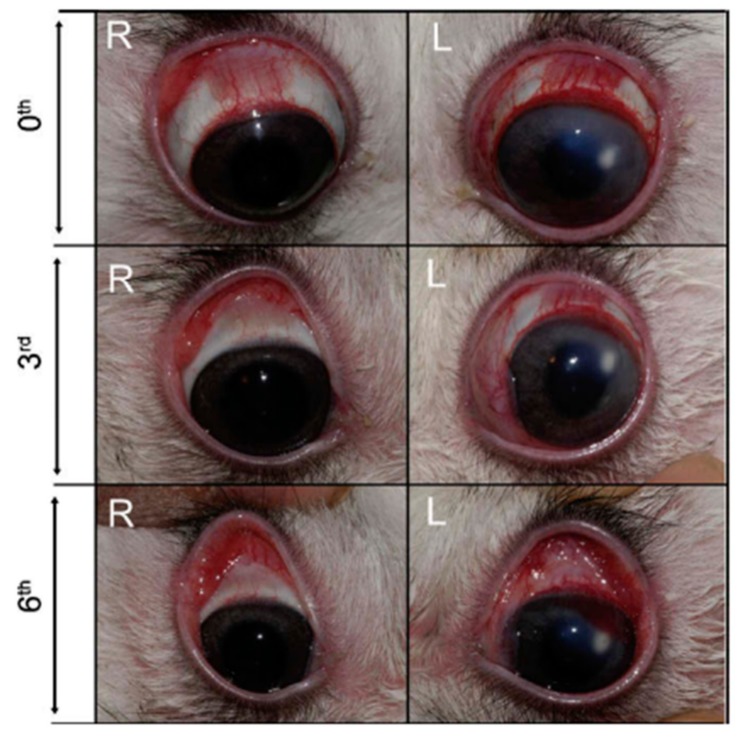
Rabbits’ eyes infected with S. aureus after 3- and 6-days treatment with bioadhesive sulfacetamide-loaded polycarbophil microspheres (right eyes, R) and sulfacetamide alone (left eyes, L). Reproduced from Sensoy et al. [55].

In order to increase the residence time of the drug, thus enhancing its efficacy, Sensoy et al. developed bioadhesive sodium sulfacetamide microspheres which were characterized both in vitro and in vivo [55]. Rabbits’ eyes with keratitis treated with microspheres obtained lower clinical scores than control ones treated with the drug alone (Figure 7). In addition, the number of viable bacteria decreased too, concluding a superior effect of the drug-loading microspheres for the treatment of ocular keratitis [55].

A last fact to take into consideration is that sulfacetamide is also considered a pregnancy category C drug. However, despite its potential risk, its use may be justified due to its benefits in some cases. It is also contraindicated in patients with sulfonamide hypersensitivity and in patients with kidney disease [53,54]. Although it is not confirmed for topical administration of sulfacetamide, systemically absorbed sulfonamides are typically excreted in breast milk and this may cause kernicterus in the nursing infants [50].

### 3.5. Sulfisoxazole

Similar to the previous drugs, sulfisoxazole (3,4-dimethyl-5-sulfanilamidoisoxazole) is another sulfonamide with a wide antibacterial range against both Gram-positive and Gram-negative organisms, that interferes with bacterial DHPS. It has been administered for treating several diseases including severe, repeated, or long-lasting urinary tract infections, meningococcal meningitis, acute otitis media, trachoma and other bacterial and parasitic infections.

Sulfisoxazole is administered orally as tablets in combination with erythromycin for the treatment of acute otitis media in children. Although various clinical studies have proven this combination to be as effective as other antibiotics given for this indication [56,57], Krause et al. showed that amoxicillin-treated patients obtained a superior clinical outcome, thus recommending its use [58]. However, in patients with a history of penicillin hypersensitivity or with ampicillin-resistant *Haemophilus influenzae* infections, this combination or trimethoprim-sulfamethoxazole (both antifolates too) might be preferred. Moreover, Perrin et al. analyzed the effect of sulfisoxazole as a chemoprophylactic for recurrent otitis media in children of different ages and concluded that its use, at least during high-risk months, may be recommended in children under six years with this condition [59].

While sulfonamides-allergic patients must avoid sulfisoxazole treatment, no relevant side effects have been reported for non-allergic patients apart from gastrointestinal disturbances. Koch-Weser et al. analyzed the adverse side effects of sulfisoxazole, sulfamethoxazole and nitrofurantoin, which are drugs with similar therapeutic indications, and conclude that although the rate of serious reactions was quite low in the three cases, the use of nitrofurantoin carries a higher risk than the other two sulfonamides [60].

The main clinical problem related to sulfisoxazole is its bioavailability. Pharmacokinetic studies demonstrated that total elimination of sulfisoxazole is achieved between 4.6–7.8 h after the dose, depending on the route of administration [61]. Thus, it is a short-acting antibacterial. However, its low solubility leads to a poor viability at the target site. Therefore, some strategies have been developed in the past few years to solve this problem by using inclusion complexes as drug delivery systems [62,63].

Moreover, although it is not related to its antibiotic effects, it is worthy to comment about the relevant off-target of sulfisoxazole: the two isoforms of endothelin receptors. Endothelins are mostly related to vasoconstriction and play important roles in both physiological and pathological conditions. More than a decade ago, Syed et al. were the first to demonstrate that sulfisoxazole was able to protect the retina in vivo from ischemic-like insults, as occurs in glaucoma, by attenuating the elevation in nitric oxide and the reduction in GABAergic neurons [64]. Later, Uchino et al. studied its effects in rats with pulmonary hypertension, a disease where the increase in circulating endothelin-1 levels contributes to disease progression and leads to right ventricle heart failure. In the study, they confirmed that sulfisoxazole-treated rats survival rate was superior to control ones when analyzing the right heart failure and/or related organs dysfunctions [65].

Lastly, sulfisoxazole has been recently linked to anti-metastasis therapies. Exosome studies have gained much popularity in recent years due to its connection with cancer progression and metastasis and drug repurposing strategies have been utilized to find exosome secretion inhibitors. Im et al. demonstrated that sulfisoxazole interference with endothelin receptor A inhibited small extracellular vesicles secretion from breast cancer cells and its administration in mouse models of breast cancer xenografts showed antitumor and anti-metastatic effects [66].

### 3.6. Co-Trimoxazole: Trimethoprim and Sulfamethoxazole

Co-trimoxazole use consists of a combined therapy that merges the antibacterial effects of two different antifolates: trimethoprim (2,4-diamino-5-(3,4,5-trimethoxybenzyl)pyrimidine) and sulfamethoxazole (3-(p-Aminophenylsulfonamido)-5-methylisoxazole). While the first one is a pyrimidine related to pyrimethamine (see epigraph 3.8) which inhibits DHFR, sulfamethoxazole is a sulfonamide, and therefore it inhibits bacterial DHPS. Inhibiting two almost concatenated reactions of the folate cycle adds a synergic effect to its combined use [67].

In co-trimoxazole therapy, sulfamethoxazole is administered five-fold more concentrated than trimethoprim, thus diminishing the possible side effects of inhibiting the human DHFR by the latest. Such administration is usually orally given but some dosage forms may be found for intravenous injection.

Although both trimethoprim and sulfamethoxazole were prescribed as monotherapies for certain indications, and some other combinations of sulfonamides with trimethoprim are available too, co-trimoxazole therapy is currently the most used antifolates-based treatment for antibacterial purposes [68]. This is mainly due to its wide spectrum of activity, being effective against both Gram-positive and Gram-negative bacteria, its great tolerability profile and its reduced cost. Moreover, this dual therapy has been shown to delay the development of bacterial resistances when compared with the administration of the same compounds in monotherapy [8].

Co-trimoxazole therapy is the most frequently prescribed treatment for urinary tract infections. It is also administered in pulmonary infections, prostatitis cases and infections caused by multidrug resistant bacteria such as *Stenotrophomonas maltophilia* and community-associated methicillin-resistant *Staphylococcus aureus* (MRSA)*,* although resistances over 50% have been recently reported, implying the urgency to rapidly confront this emerging clinical problem [8]. Furthermore, Bowen et al. recently reviewed the role of co-trimoxazole for treating skin and soft tissues infections and highlighted its effectiveness, even in group A *Streptococcus*, which has always been very controversial [69]. Moreover, drug repurposing has focus on co-trimoxazole as a potential treatment for drug-resistant tuberculosis. Although some limitations have been proposed such as the number of resistant strains tested or possible drug interactions, it seems a promising alternative to take into consideration in the future [67].

In addition, it can be used as a prophylactic for opportunistic infections in HIV-positive patients, whose CD4^+^ T cells are lowered, against pathogens like *Pneumocystis jirovecii* or *T. gondii* [70]. However, it is not more effective or safer when compared with other treatments for HIV-associated cerebral toxoplasmosis, thus it cannot be considered as the preferred treatment [71]. Furthermore, it can be used as an antimalarial [70].

This treatment is usually well-tolerated and the side effects are uncommon, but includes cutaneous eruptions and exanthemas as well as hyperkalemia and hyperglycemia, especially in patients with renal insufficiency and due to its interactions with other drugs. Nevertheless, some predictable and life-threatening side effects have been previously reported regarding hematological effects, due to disturbances in the folic acid cycle. Ho and Juurlink conducted an extensive research on the side effects produced by co-trimoxazole covering clinical reports between 1980 and 2011 and they classified and analyzed, reviewing their frequency and postulated risk factors [70].

Regarding gestational issues, co-trimoxazole is a pregnancy category D drug, meaning that there are human studies where severe side effects have been observed. The majority are related to congenital malformations including neural tube defects, cardiovascular malformations or urinary tract defects [72,73]. Sometimes it is used as an alternative to treat asymptomatic bacteriuria during pregnancy but only in the six first weeks of pregnancy [74] and always folic acid supplementation is co-administered to reduce the associated side effects on the foetus.

### 3.7. Dapsone (Part I) as an Antibiotic

Dapsone (4,4′-diaminodiphenyl sulfone) is another sulfonamide which exerts its bactericidal function by competing with PABA on the active site of the bacterial DHPS. It has been largely prescribed as an antibacterial for treating leprosy caused by sensible *Mycobacterium leprae* strains, being part of several combined therapies [75]. However, the appearance of resistant strains has forced scientists to start looking for new alternatives. The molecular basis of dapsone resistance was elucidated thanks to the use of computational modeling analyzing the interaction between the drug and the enzyme [76], and this has been used to synthesize novel dapsone derivatives that could be further use as a newer antileprosy therapy [77].

Dapsone is also used as a prophylactic drug for *P. jirovecii*-caused pneumonia in patients who cannot tolerate co-trimoxazole therapy for some reason. However, this dual therapy remains as the most effective treatment for preventing this kind of fungal infections, thus being recommended as a first-line drug [78,79]. Furthermore, it has been marketed as gels for topical administration to treat some forms of acne thanks to its combined antibacterial and anti-inflammatory mechanisms [80].

There are two major concerns associated to the use of dapsone in the clinical setting. The first one is related to its toxicity. In contrast to other sulfonamides which are highly well-tolerated, adverse reactions to dapsone occur frequently. The most usual side effects are hematological, including hemolysis (most common), methemoglobinemia and sulfhemoglobinemia, agranulocytosis (rare but fatal), aplastic anemia (rare but fatal) or red cell aplasia. In addition, some cardiovascular, respiratory, pancreatic and even psychiatric events have been reported to be caused by dapsone treatment [75,79]. Moreover, its prolonged administration has been shown to produce a condition termed as “dapsone syndrome” and which is characterized by fever, hepatitis, leukopenia, exfoliative dermatitis, lymphadenopathy and mononucleosis, arising within the first six weeks of treatment [81].

Furthermore, dapsone is considered a pregnancy category C drug. Although an increased risk of congenital abnormalities has never been reported when administered during pregnancy, some hematological side effects does have. This is especially important regarding leprosy because pregnancy may be a trigger for this disease due to the changes in the immune system. As the 20% of children born from mothers with leprosy may experience leprosy by puberty, it is of pivotal relevance treating women during pregnancy [79,82]. It is also indicated for treating HIV-infected pregnant women with encephalitis caused by *T. gondii*.

The second major limitation of dapsone is its low solubility, which in the end results in reduced therapeutic indexes and a higher microbial resistance. For this reason, new approaches have been investigated like the one proposed by Chaves et al. They developed a novel pH-responsive dapsone-loaded chitosan-based hydrogel which was designed as a potential oral formulation for the treatment of leprosy. The hydrogel was able to load up to 60% of the 24 mg *per* formulation while achieving the controlled release of the drug [83].

### 3.8. Antimalarial Antifolates: Proguanil and Pyrimethamine/Sulfadoxine

Finally, a last section on the use of antifolates as antimalarial agents will be reviewed. Malaria is a parasitic infection caused by different species of *Plasmodium* such as *P. falciparum* (the deadliest) or *Plasmodium vivax* (the most frequent) with more than 500 million infections worldwide every year [3]. Nowadays, first-line treatments are centered on the use of either artemisinin-based combination therapies or chloroquine administration. The mechanisms of actions of these drugs differ from antifolates-based therapies, whose use has been limited to resistant strains to these first-line treatments [84].

On the contrary to bacteria, many strains of *P. falciparum* can internalize exogenous folate and metabolize it. Therefore, inhibiting folate de novo biosynthesis is not as effective as in the previous cases for both treatment and prophylaxis. For this reason, combined therapies that act synergistically in different steps of the route are preferred. Briefly, some of these combinations include chlorproguanil with dapsone (LapDap), pyrimethamine with sulfadoxine (Fansidar) and pyrimethamine with dapsone (Maloprim) [3,85].

Proguanil (1-(*p*-chlorophenyl)-5-isopropylbiguanide) and its derivatives. It is one of the most common choices among the different options to treat chloroquine-resistant strains and as prophylaxis. It is administered in combination with atovaquone, which selectively inhibits *Plasmodium* mitochondrial electron transport at the level of the cytochrome bc1 complex, while proguanil inhibits DHFR, thus resulting in a disrupted metabolism [85]. In addition, a therapy combining chlorproguanil with dapsone has been designed to minimize the appearance of resistances to these compounds [86].

Pyrimethamine/sulfadoxine. In this dual therapy, DHPS is inhibited by the sulfonamide while pyrimethamine (2,4-Diamino-5-(4-chlorophenyl)-6-ethylpyrimidine) selectively inhibits plasmodial DHFR. These sequential enzymatic suppression finally results in a synergistic effect much greater than an additive effect [3]. However, the rate of reported hypersensitivity reactions to the drug was too high and it was not further recommended as a prophylactic therapy, although it is still used in some parts of Africa [85].

Pyrimethamine/Dapsone. This last antimalarial combination has been controversial due to the serious side effects which have been associated with its administration like bone marrow suppression. For this reason, other safer antimalarials are preferred [85].

Regarding plasmodial folate metabolism, it has some key features such as bifunctional enzymes or the inability to salvage preformed pyrimidines that might allow the rational design of new drugs. However, little work has been done in this sense. Nzila et al. excellently reviewed this topic in depth, highlighting multiple interesting approaches to conventional drugs [86].

## 4. Antifolates as Immunomodulating Agents

Apart from their use as antibiotic agents, some immunomodulatory properties have been reported for a few antifolates. In this review, the term immunomodulation will be used to refer any biochemical process that leads to modify the immune system in a desired way with therapeutic interest, including, for instance, anti-inflammatory properties or immunosuppressive effects. In this context, in contrast to their well-stablished antibacterial mechanisms of action, the specific immunomodulatory effects of these drugs are not known in detail and much more research is needed. However, these antifolates are used in the clinical setting for different immune-related indications due to their efficacy and to the lack of other appropriate alternatives.

### 4.1. Dapsone (Part II) as an Immunomodulator

Main characteristics of dapsone have already been commented earlier in this review when addressing its antibacterial use. However, apart from a treatment for leprosy, there are two labeled and some off-label indications for which dapsone is prescribed due to its immunomodulating properties. Among these indications, many skin diseases with a component of abnormal infiltration of neutrophils and eosinophils are found such as dermatitis herpetiformis and acne vulgaris (the labeled ones), linear IgA dermatosis, pustular psoriasis, bullous systemic lupus erythematosus, pyoderma gangrenosum, Sneddon-Wilkinson disease or Sweet’s syndrome among others. It also has been administered in rheumatoid arthritis patients and for treating brown recluse spider bites [79].

The etiology of many of these diseases is yet to be determined but those which are known are very different from each other. Therefore, it seems that dapsone does not affect the initial pathologic process, but it regulates the effector mechanisms. Neutrophils and eosinophils are found to play a central role in these mechanisms as reviewed by Zhu et al. and, more recently, by Wozel & Blasum. Only the main and confirmed anti-inflammatory mechanisms of dapsone are summarized below. For extended literature, these two reviews are highly recommended [79,87].

Antioxidant effects of dapsone can be largely attributed to the inhibition of reactive oxygen species (ROS) production rather than a scavenging activity of the molecule as it has been historically proposed. Dapsone has been demonstrated to suppress N-formyl-methionyl-leucyl-phenylalanine-induced production of extracellular O_2_^-^. In addition, dapsone has been found to irreversibly inactivate both neutrophils’ myeloperoxidase and eosinophils’ peroxidase at concentrations similar to serum levels achieved with typical doses used in clinic, inhibiting the production of toxic substances (e.g., hypochlorous acid) and elongating the median lifespan of *Caenorhabditis elegans.*

Effects on eicosanoid production. Dapsone inhibits choline phosphotransferase and methyltransferases I and II, which are enzymes related to arachidonic acid liberation after cell activation by different stimulus. Thus, it has a negative effect on the subsequent synthesis of arachidonic acid derivates such as prostaglandins and leukotrienes. Moreover, dapsone inhibits 5-lipoxygenase, which is the enzyme that starts the reactions cascade to synthesize leukotrienes from arachidonic acid, and maybe some other enzymes of the pathway, reducing the inflammatory effects derived from these compounds. It is believed that its suppressive effect on the synthesis of leukotriene C_4_ can be related with its corticosteroid-sparing effects.

Effects on chemotaxis. Dapsone has been demonstrated to inhibit chemotaxis or not depending on the stimuli applied (e.g., it does not inhibit leukotriene B_4_-induced chemotaxis). When it does inhibit neutrophils chemotaxis, dapsone interferes with the action or activation of inhibitory-type G-proteins which would transduce the chemotactic signal in normal conditions. Therefore, neutrophils recruitment to inflamed zones is reduced, adding a secondary anti-inflammatory effect by avoiding the release of other pro-inflammatory mediators in these areas [79,87].

Ultimately, all these mechanisms act cooperatively and make dapsone an unique drug for the treatment of several diseases with an important inflammatory component. Moreover, in the last decade, some neuroprotective effects have been associated to dapsone due to its wide range of antioxidative and anti-inflammatory properties. In fact, interleukin 8 (IL-8), which is highly influenced by ROS and of pivotal importance in many neurologic diseases, has been found to be subexpressed and blocked by dapsone. Therefore, dapsone is being currently investigated as a possible drug for IL-8-mediated diseases [87].

Nevertheless, dapsone as an immunomodulator has the same clinical problems as if it was used as an antibiotic. To overcome this, some derivatives have been synthesized with different purposes, including polymers which uses dapsone-derivatives as monomers. Rojo et al. developed dapsone polymer conjugates of 2-hydroxyethyl methacrylate (HEMA) and dapsone methacrylamide which demonstrated not only to maintain the anti-inflammatory properties of dapsone but also increasing them synergistically with the addition of the HEMA monomer. HEMA incorporation reduced the cytotoxicity of the co-polymers too, which enables its exploitation for future biomedical applications [88].

### 4.2. Methotrexate

Methotrexate is a multi-functional drug that is administered in high doses for treating diverse types of cancer (specially leukemias and lymphomas) and in lower doses for auto-immune diseases such as psoriasis or rheumatoid arthritis, due to its anti-inflammatory and immunosuppressive effects [89]. Although additionally having antibacterial properties, the high structural similarity between bacterial and human DHFRs makes it inappropriate for being administered as an antibiotic agent.

Regarding its immunomodulatory role in rheumatoid arthritis, methotrexate is considered as a disease-modifying antirheumatic drug (DMARD), meaning that it slows down disease progression. It is indicated as a first line treatment as a monotherapy due to its effectivity and reduced cost. However, despite having been used since the 1980′s, its actual mechanism of action is still under debate [89,90].

Contrary to previous antifolates, methotrexate inhibits not only one, but three different enzymes related to folate cycle: DHFR, TS and ATIC. These inhibitions are of pivotal importance when administered in high doses for treating cancers, but it seems not to be the principal mechanism underlying the clinical benefits achieved in rheumatoid arthritis patients. This is because in these cases it is usually co-administered with folate to reduce its adverse effects and this co-administration does not result in a decrease in clinical efficacy. Therefore, other pathways must be involved.

**Figure 8:**
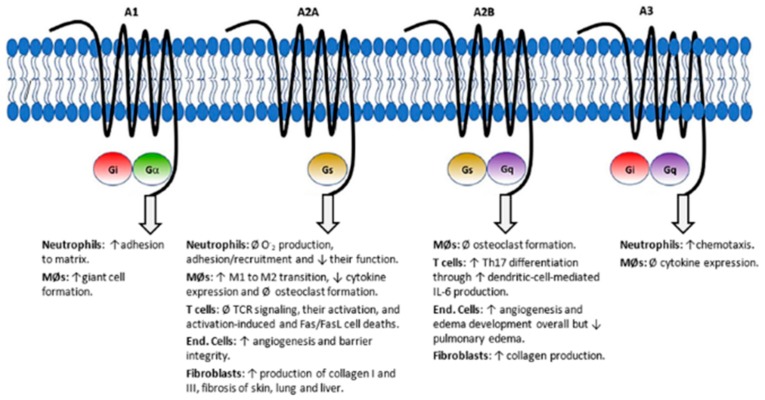
Adenosine receptors and signaling pathways towards anti-inflammatory phenotype macrophage polarization. Abbs.: ↑ = increase, ↓ = decrease, Ø = inhibition, End. = endothelial, MØs = macrophages. Reproduced from Friedman & Cronstein [90] under Creative Commons Attribution License (CC BY 4.0).

Currently, the most accepted hypothesis focuses on the potentiation of adenosine signaling. Adenosine is a well-known paracrine immunomodulating molecule which acts through four G-protein-coupled receptors (GPCRs; A1, A2A, A2B and A3), having different responses depending on the activated GPCR. ATIC inhibition by methotrexate leads to an increase in extracellular adenosine and it is currently accepted that the observed anti-inflammatory effects are mainly caused by the activation of the A2A receptor. In fact, an overexpression of these receptors have been reported in immune cells of rheumatoid arthritis patients, indicating a major role in the mechanism of action of this drug [89,90]. Macrophage polarization towards an anti-inflammatory phenotype and inhibition of T cells and neutrophils activation are among the resulting effects of A2A signaling (Figure 8) [90,91].

Other mechanisms which have been studied over the years and which may be secondary involved have been extensively reviewed by Brown et al. and, more recently, by Friedman & Cronstein [89,90]. On the one hand, methotrexate additionally induces T cell apoptosis which may be one of the causes for an increase in ROS. Other possible explanations for this increment may be related to the uncoupling of endothelial nitric oxide synthase due to the dysregulation of DHFR or to the inhibition of polyamines which are ROS scavengers. In any case, this ROS accumulation leads to T cell apoptosis, which positively feeds-back the cycle and ultimately diminish the inflammation via T-cell immunosuppression.

On the other hand, by targeting different immune cell types, methotrexate alters the cytokine profile towards a more anti-inflammatory environment. Many studies have focused on analyzing its effect on neutrophils, mast cells and CD4^+^ T cells but only a few have centered on CD8^+^ T cells. In this regard, Sandhu et al. have recently reported that methotrexate decreases the CD8^+^ IFNγ^+^ T cells population while marginally increases CD8^+^ IL17^+^ T cell population in rheumatoid arthritis patients. In addition, circulating levels of IL-2, IL-10 and IL-17 were diminished after methotrexate administration [92]. However, although some hypotheses have been proposed (e.g., altering NF-κB pathway), the direct molecular mechanisms by which cytokine profiles are modified by methotrexate are still unclear [89].

Finally, rheumatoid arthritis is characterized not only by the inflammation but also by the joint destruction that it causes. In this sense, methotrexate has been proved to reduce the serum concentrations of different types of metalloproteinases, which are responsible of both bone and cartilage erosion. In addition, by adenosine signaling, methotrexate inhibits macrophage differentiation towards osteoclasts, thus reducing bone resorption [89]. Nevertheless, achieving a successful regeneration of the eroded tissues is particularly difficult because of the residual inflammatory environment, which is not high enough to enable disease progression, but which largely delays the repair of these tissues. To overcome this, we have designed novel methotrexate derivatives bearing different divalent cations such as strontium, zinc or magnesium, which can be easily synthesized, and which have been demonstrated to increase glycosaminoglycans deposition by chondrocytes to promote cartilage regeneration [93,94].

### 4.3. Sulfasalazine

Sulfasalazine (2-hydroxy-5-((4-((2-pyridinylamino)sulfonyl)phenyl)azo)benzoic acid) is a modified sulfonamide composed of one sulfapyridine and one 5-aminosalacyclic acid (5-ASA) moieties covalently linked by an azo bond. While the first one was used in the past as a treatment for dermatitis herpetiformis until its commercialization abandonment in the 1990′s, 5-ASA was largely believed to be the only responsible for the immunomodulating effects of sulfasalazine, which would be later proved wrong.

Nowadays, sulfasalazine is commonly used for the treatment of inflammatory bowel diseases such as Chron’s disease or ulcerative colitis and other autoimmune diseases like ankylosing spondylitis or psoriasis. In addition, it is classified as a first line DMARD and some rheumatologist prefer its early use to other alternatives such as methotrexate or bucillamine because of its demonstrated efficacy in slowing disease progression [95]. Its therapeutic effects are related with both immunomodulating and anti-inflammatory properties of the drug. However, its mode and site of action are not fully comprehended. The most relevant findings related to these effects are commented below.

Effects on soluble mediators of inflammation. Sulfasalazine has been demonstrated to inhibit the release of various interleukins such as IL-1, IL-2, IL-6 and IL-12 as well as the tumor necrosis factor alpha (TNF-α) by different cellular types, although the responsible mechanism has not been elucidated yet [95,96].

Effects on immune cells. A suppressive effect has been shown in B cells after sulfasalazine treatment *in vitro*, inhibiting IgM and IgG production, including IgG anti-DNA antibodies which are known to play an important role in the pathophysiology of rheumatoid arthritis. On the other hand, sulfasalazine inhibits T-cells proliferation and prevents their stimulation by dendritic cells while promoting their apoptosis by a caspase-independent mechanism which involves the mitochondrial-nuclear translocation of the apoptosis-inducing factor (AIF) [97]. It also reduces neutrophils migration to inflamed regions by inhibiting chemotaxis signals and promoting neutrophils apoptosis [95].

Anti-inflammatory mechanisms. Like other DMARDs, the main anti-inflammatory effects of sulfasalazine seem to be due to the increase in extracellular adenosine that it generates. This increase in extracellular adenosine is mediated by the extracellular activity of ecto-5′-nucleotidase (CD73), which uses adenine nucleotides as substrate for the reaction [98]. Some other anti-inflammatory effects have been shown such as the inhibition of the extracellular release of proinflammatory secretory phospholipase A_2_ or the inhibition of leukotrienes and prostaglandin E_2_ syntheses [99]. However, these effects have always been very minor when compared with adenosine mechanism [95].

In addition, sulfasalazine has been shown to scavenge ROS, which could be another anti-inflammatory mechanism, but these reactions also generate toxic free radicals which are in part the cause of the side effects of the drug [96,100]. Most of these side effects are mild such as headache or nausea, but some others are more severe like infertility, nephro- and hepatotoxicities, pancreatitis or allergies [96]. Interestingly, in contrast to the toxicity observed in children and adults, its use is safe in pregnancy. Recently, Brownfoot et al. demonstrated that sulfasalazine is able to reduce the secretion of soluble endoglins and other proteins related to endothelial dysfunction while upregulates the secretion of placental growth factor (PlGF). Thus, taking advantage of its safety under pregnancy, it could be used in the future for the treatment of preeclampsia, a complication with no available medical treatment [101].

## 5. Development of Resistances to Antifolates

After their discovery in the 1930s, sulfonamides use rapidly gained popularity, giving rise to the era of the safe systemic antibiotic therapies. However, its associated environmental stress obliged bacteria to evolve through different mechanisms in order to adapt to the new antibiotics [102]. Analogous mechanisms have been described due to the use of antifolates as antiparasitic, chemotherapeutic and immunomodulating agents. Commenting every possible mutation which has occurred in every specie or enzyme is beyond the scope of this review. Instead, the molecular mechanisms underlying these resistances will be addressed and some examples of interest will be discussed.

**Figure 9:**
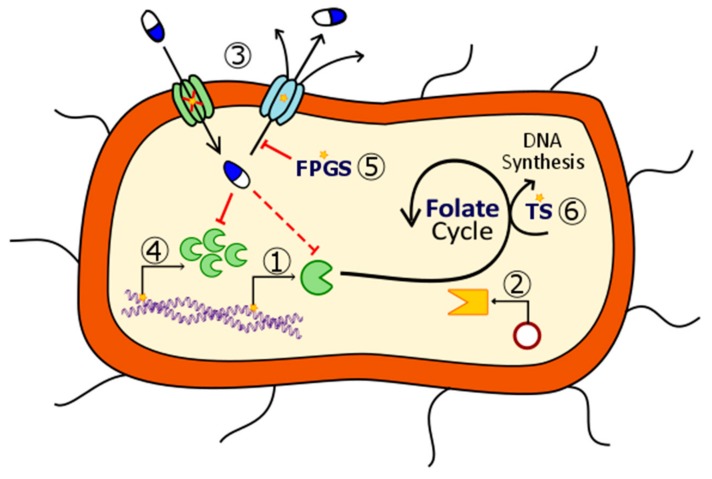
Resistance mechanisms against antifolates. Stars indicate possible mutations associated with the appearance of resistance against antifolates. Some mechanisms apply to eukaryotic cells too. Each number indicates the resistance mechanism described in the text: 1. Mutations affecting the genes that code the antifolates-targeted enzymes; 2. Emergence of novel resistant isoforms of the antifolates-targeted enzymes; 3. Reduction in cell permeability and increase in efflux proteins; 4. Overexpression of target enzymes; 5. Deregulation of polyglutamation; and 6. Thymine auxotrophy.

Regarding their origin, antifolates resistances can be divided into intrinsic, when due to the characteristics of the target organisms (microorganisms or eukaryotic cells), they are immune to the drug, or acquired, caused by environmental pressure. On the other hand, antifolates can be classified also regarding where the change that originates the resistance occurs. For instance, resistance may be due to a modification in the drug mediated by the target organism, it also may be due to a change in the target organism, or, finally, it can be due to a change in the environment too. However, it seems that only the changes in the target organisms are responsible of the long-term resistances [103]. Thus, only these molecular mechanisms are detailed below and schematized in Figure 9.

1. Mutations affecting the genes that code the antifolates-targeted enzymes such as DHPS or DHFR. In these cases, the affinity of the inhibitors for the enzyme is diminished, which is confirmed by the increased in the affinity constant, *K*_i_. Thus, a higher concentration of the drug must be employed to obtain the same therapeutic effect [104]. This mechanism is typical of Gram-positive bacteria against sulfonamides. These mutations can be diverse. For example, in *Escherichia coli* single punctual mutations in the *folP* gene have been reported to avoid the inhibitory function of sulfonamides by affecting the binding sites of these drugs and the DHP-PPi (binding sites that are highly conserved among species) [105,106]. On the contrary, in *Staphylococcus* spp. mutants have been found where the accumulation of punctual mutations resulted in a divergence of 14 amino acids when compared with sensible enzymes, and these residues were located over the surface of the enzyme which difficulted the results interpretation for determining the molecular mechanism of resistance [106,107]. Moreover, larger mutations have been reported in the *Neisseria meningitidis folP* gene, giving rise to mosaic genes, which codified enzymes whose C- and N-terminal ends belong to sensible genes while the catalytic part is identical to a resistant variant [106,108].

2. Emergence of novel resistant isoforms of the antifolates-targeted enzymes. In contrast to the previous mechanism, this one is more typical from Gram-negative bacteria such as *Enterobacter* spp. Some examples are the two well-known genes that mediate sulfonamide resistance, *sul1* and *sul2*, which were extensively characterized in the past. The first one is present on class 1 integrons while *sul2* is found on plasmids [109]. Interestingly, only these two genes are the ones responsible for the worldwide sulfonamide resistance in Gram-negative bacteria. It has been proposed that this may be due to their location in easily disseminating plasmids or that the enzyme needs a very constrained atomic disposition to catalyze the reaction, binding the substrate while rejecting the inhibitor. Thus, not many conformations are allowed [106]. A third plasmid-borne gene, *sul3*, was later identified in farm animals. Similar examples are known for trimethoprim-resistant DHFR enzymes but, in this case, there are more than 20 resistant variants which move from one organism to another on class 1 and class 2 integrons [109].

3. Reduction in cell permeability and increase in efflux proteins. Both mechanisms seek to diminish the drug concentration inside the target cell, whether by avoiding its entrance or by expelling them to the outside. Regarding the permeability issue, Gram-negative bacteria possess porins in their membrane, which allow the interiorization of small molecules (including antibiotics). Although mutations in these proteins have been reported to cause resistances to other antibacterials and some studies using antifolates suggest that this could be another mechanism of resistance for these drugs, there are no conclusive data to confirm it [103,104,110,111,112]. As many of these articles propose, it would be necessary to carry out additional studies using, for example, radiolabeled drugs to confirm this mechanism, which could act in synergy with the previously commented ones. Regarding the efflux proteins, their associated-resistance mechanism was described for tetracyclines long ago and like the permeability issue, there are no consistent studies to infer an antifolate-based antibacterial mechanism [109]. However, it is quite usual in eukaryotic organisms and it has been described, for instance, for methotrexate as a chemotherapeutic agent [113].

4. Overexpression of target enzymes. Folate cycle is a thoroughly regulated system to ensure the optimal concentrations of all the intermediates at every moment. Therefore, if the levels of expression of one enzyme increase, this can lead to the deregulation of the pathway. In eukaryotic cells, this process of enzyme overexpression is the result of gene duplication [109]. In this respect, overexpression of target enzymes results in a mechanism of resistance in both bacterial (e.g., trimethoprim resistance described in *E. coli*) and eukaryotic cells (e.g., methotrexate resistance in cancerous cells) [110,113].

5. Deregulation of polyglutamation. A decrease in the levels of expression of FPGS or a reduction of its activity by punctual mutations leads to an impairment in the polyglutamation degree of classical antifolates. Like these ones are not used for treating bacterial infections, this resistance mechanism is typical of human cells and parasites [113].

6. Thymine auxotrophy. There is one interesting mechanism that confers resistance to both sulfonamides, trimethoprim and its combination and it is due to disfunction of only one enzyme: thymidylate synthase. As early commented in this review, the intracellular THF pool largely depends on the salvage pathway mediated by TS, which uses N^5^,N^10^-Methylene THF to methylate dUMP to dTMP. These organisms cannot synthesize dTMP de novo, needing to absorb it from the outside and therefore being termed as thymine-requiring mutants (TRMs). For this reason, TRMs adapt their metabolism to reduce the requirements of THF and this is what confers the resistance to all these drugs. However, TRMs do not suppose a major concern due to their reduced virulence and low incidence. They have been found in human infections where co-trimoxazole treatment had previously failed but their role as pathogens seems very limited [103].

Finally, there are two more issues of interest that it is important to mention when addressing antibiotic resistances. The first one is the cross-resistance among drugs. It is essential to highlight that resistance to one sulfonamide confers resistance to all sulfonamides and analogously for DHFR inhibitors [110]. This has been the major disadvantage since their discovery due to the resistances that have been generating over time. For instance, some reports estimated that co-trimoxazole resistance to hospital acquired-MRSA infections exceed the 50% of the cases although both frequency and mechanisms of resistance highly vary attending geographic and cohort issues [8].

And in this context, it would be worth considering if the abandonment of the antifolates-based antibacterial therapy could result in the disappearance of these resistance mechanism in the long-term. As previously commented, when mutations occur in *folP* gene and the *K*_i_ for the sulfonamides increase, a resistance mechanism is developed. However, when this happens, the *K*_m_ of the natural substrate of DHPS (PABA) also increases, meaning a reduction in the efficiency of the enzyme [106,114]. Thus, it could be assumed that being in disadvantage when compared to sensible strains, resistant mutants might disappear in the long-term. Nevertheless, as extensively discussed by Ola Sköld, this is not likely to happen because the resistant strains will accumulate new mutations that compensate the side effects of the resistance development [106].

## 6. Current Experimental Compounds and Future Trends on Antifolates Research

All these limitations that resistances to antifolates pose have obliged scientist to look for new solutions facing the future. To conclude this review, a brief comment on the two more relevant antifolates-as-antibiotics-based research lines will be made and particular emphasis will be made on iclaprim, the only new-generation antifolate which has reached the market.

Novel inhibitors of DHPS. As early commented, sulfonamides bind to the PABA pocket within DHPS, avoiding the entrance of this substrate. However, there is a second substrate in this reaction: the DHP-PPi. It has its own binding site and, in recent years, scientists’ attention has been redirected towards this novel potential target. While the classical PABA pocket was highly flexible, allowing the creation of several different compounds, it also gave rise to a higher probability of point mutations that led to the current existing resistances. In contrast, DHP-PPi pocket is much more conserved and rigid and, therefore, it seems that generation of new resistances would be much more difficult. Nevertheless, one of the principal drawbacks of targeting this site is the actual structure of its substrate. Being the DHP-PPi highly planar, DHP-PPi-mimicking inhibitors would be poorly soluble and tunable [8,115].

Some alternatives have been proposed during the last years as early steps for future drug development [116,117]. This is the case of the recent work of Dennis et al., where they not only synthesized derivatives of 8-mercaptoguanine (a pterin-like compound) that inhibit DHPS by targeting DHP-PPi pocket with great potency (sub-micromolar affinities) but also inhibited other enzymes of the folate cycle such as 6-hydroxymethyl-7,8-dihydropterin pyrophosphokinase, which catalyzes the previous reaction to DHPS-mediated one [118]. All in all, rational drug design in combination with a greater knowledge on specific DHPS structures seems to be a great alternative to conventional methods.

**Figure 10:**
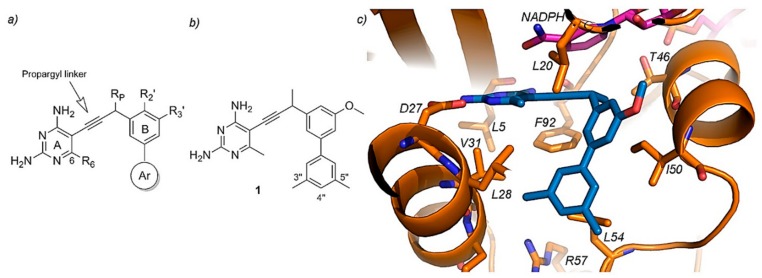
Propargyl-linked antifolates. (**a**) General scaffold with the diaminopyrimidine ring (A), phenyl ring (B) and aryl ring (Ar) along with possible positions for substitutions (R6, RP, R29 and R39). (**b**) A concrete example of a propargyl-linked antifolate, a biphenyl one, with labeled atom positions (compound 1). (**c**) Active site of a resistant DHFR, showing active site residues (orange), NADPH (magenta) and compound 1 (blue). Reproduced from Viswanathan et al [120].

Propargyl-Linked Antifolates. The other approach which has also gained popularity in the recent years focuses on targeting DHFRs, being useful for both Gram-positive and Gram-negative bacteria, including TMP-resistant species too. Propargyl-linked antifolates consists of a highly conserved diaminopyrimidine ring linked through an acetylenic group to a biaryl system which allows the tunability of the compounds as shown in Figure 10 [119,120]. The success of these drugs on the resistant DHFR enzymes is based on their shared action mechanism, where the diaminopyrimidine ring binds a conserved acidic residue of the enzyme allowing the rest of the molecule to penetrate into the active site [119]. These novel drugs have achieved inhibitory potencies in the nanomolar scale and therefore are promising candidates for antibiotic purposes [8,115].

Lastly, although these novel approaches seem promising, they are far from reaching clinical trials in the short term and undesired side effects could appear. In this sense, it is worth mention the case of iclaprim, a trimethoprim derivative which was designed to closely contact the hydrophobic regions of the substrate binding pocket of DHFR. When compared with trimethoprim, iclaprim achieved inhibitions of DHFR 20-fold greater, and was useful against trimethoprim-resistant DHFRs [121].

Iclaprim was firstly synthesized and patented in 1997 by Hoffmann-La Roche and, although it showed promising results during the in vitro preclinical phase, after several phase III clinical trials it did not achieve a superior efficacy when compared with the existing therapies, being dropped in 2008 [8]. However, in 2015, another biopharmaceutical company (Motif Bio) carried out additional clinical trials to evaluate iclaprim in acute bacterial skin and skin structure infections, obtaining positive results this time. Therefore, iclaprim is an excellent example of rational drug design and the last one to have been commercialized [121].

## 7. Conclusions

Antifolates as antibacterial, antiparasitic or immunomodulating agents have been largely used since their first discovery in the 1930s. Much research has been conducted since then to finally be able to know their target enzymes, their modes of action and many other critical factors which allow clinicians prescribe them optimally. However, their widespread use soon resulted in high percentages of resistant strains to these compounds, leading to an urgent need to develop new antibiotic therapies and look for additional targets. In this sense, all this previous research now enables scientist to rationally design new antifolate-based inhibitors.

Regarding currently clinically prescribed antifolates, it seems that these drugs might have an “expiration date” sooner or later due to all the developed resistance mechanisms described above. Until this point, the administration of combined therapies and antifolate delivery systems which allow minimal drug concentrations while increasing their residence times are recommended. On the other hand, some novel applications may be discovered by drug repurposing methods as described for sulfisoxazole or co-trimoxazole.

## Figures and Tables

**Table 1:** Summary of the reviewed antifolates as antibiotic agents, including their modes of action, indications, mode of administration and toxicity issues.

Drug		Mode of Action	Indication	Administration	Toxicity	References
**Mafenide** 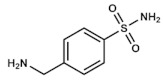		Unknown	Severe burns wounds	Topical: creams or powders for solution	Metabolic acidosis, allergies	[31,32,33,34,35,36,37,38]
**Silver Sulfadiazine** 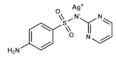		DHPS inhibitor + silver biocide effect	Burns and ulcers	Topical: creams or hydrogels	Allergies, sensitivity to silver or propylene glycol. Rare: hemolysis, argyria or pseudo-eschar formation	[32,33,39,40,41,42]
**Sulfadiazine** 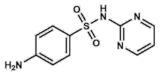		DHPS inhibitor	Urinary tract infections, otitis media, encephalitis, meningitis. Prophylaxis for rheumatic fevers. Parasitic infections: malaria and toxoplasmosis	Oral: tablets	Allergies and gastrointestinal upset. Rare: encephalopathies, renal failure, nephrolithiasis	[43,44,45,47,48,49]
**Sulfacetamide** 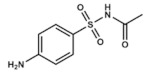		DHPS inhibitor	Skin, ocular and urinary tract infections	Ophthalmic: solution/drops Topical: ointment or lotions Vaginal: creams	Rare: mild cutaneous reactions. Category C drug in pregnancy	[50,51,52,53,54,55]
**Sulfisoxazole** 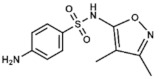		DHPS inhibitor	Severe, repeated, or long-lasting urinary tract infections, meningococcal meningitis, acute otitis media, ocular infections, etc.	Oral: tablets or suspensions with or without erythromycin	Rare. Allergies, gastrointestinal disturbances	[56,57,58,59,60,61,62,63,64,65,66]
**Co-trimoxazole**	**Sulfamethoxazole** 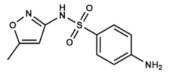	DHPS inhibitor	Bacterial bronchitis, prostatitis and urinary tract infections.	Oral: tablets of both compounds (co-trimoxazole)	Allergies, nausea, vomiting, diarrhea and hematologic alterations. Category D drug in pregnancy	[8,67,68,69,70,71,72,73,74]
	**Trimethoprim** 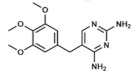	DHFR inhibitor				
**Dapsone** 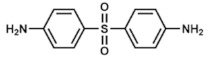		DHPS inhibitor	Leprosy. Prophylaxis for co-trimoxazole-resistant P. jirovecii infections. With pyrimethamine: malaria	Oral: tablets or suspensions Topical: gels	Hemolysis (usually mild), dapsone syndrome, gastrointestinal upset. Category C drug in pregnancy	[75,76,77,78,79,80,81,82,83]
**Antimalarial Agents**	**Proguanil** 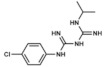	DHFR inhibitor	Malaria treatment and prophylaxis	Oral: tablets	Hypersensitivity reactions, bone marrow suppression	[3,84,85,86]
	**Pyrimethamine** 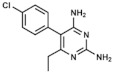	DHFR inhibitor				
	**Sulfadoxine** 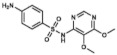	DHPS inhibitor				

## Abbreviations

AICARFT	Aminoimidazole-4-carboxamide ribonucleotide formyltransferase
AIF	Apoptosis-inducing factor
ATIC	Aminoimidazole-4-carboxamide ribonucleotide formyltransferase/inosine monophosphate cyclohydrolase
DHF	Dihydrofolate
DHFS	Dihydrofolate synthase
DHFR	Dihydrofolate reductase
DHP-PPi	Dihydropteroate pyrophosphate
DHPS	Dihydropteroate synthase
DMARD	Disease-modifying antirheumatic drug
dUMP	2′-deoxyuridine-5′-monophosphate
dTMP	2′-deoxythymidine-5′-monophosphate
FDTS	Flavin-dependent thymidylate synthase
FPGS	Folylpoly-γ-glutamate synthetase
GARFT	Glycinamide ribonucleotide formyltransferase
GGH	γ-glutamyl hydrolase
GPCR	G-protein-coupled receptor
HEMA	2-hydroxyethyl methacrylate
IL	Interleukin
MRSA	Methicillin-resistant *Staphylococcus aureus*
PABA	*p*-aminobenzoic acid
PPi	Pyrophosphate
ROS	Reactive oxygen species
THF	Tetrahydrofolate
TNF-α	Tumor necrosis factor alpha
TRMs	Thymine-requiring mutants
TS	Thymidylate synthase
